# Evolution of hypoxia and hypoxia-inducible factor asparaginyl hydroxylase regulation in chronic kidney disease

**DOI:** 10.1093/ndt/gfad075

**Published:** 2023-04-24

**Authors:** Anna Faivre, Romain Dissard, Willy Kuo, Thomas Verissimo, David Legouis, Grégoire Arnoux, Carolyn Heckenmeyer, Marylise Fernandez, Matthieu Tihy, Renuga D Rajaram, Vasiliki Delitsikou, Ngoc An Le, Bernhard Spingler, Bert Mueller, Georg Shulz, Maja Lindenmeyer, Clemens Cohen, Joseph M Rutkowski, Solange Moll, Carsten C Scholz, Vartan Kurtcuoglu, Sophie de Seigneux

**Affiliations:** Department of Medicine and Cell physiology and Metabolism, University of Geneva, Geneva, Switzerland; Service of Nephrology, Department of Medicine, Geneva University Hospitals, Geneva, Switzerland; Department of Medicine and Cell physiology and Metabolism, University of Geneva, Geneva, Switzerland; Institute of Physiology, University of Zurich, Zurich, Switzerland; National Centre of Competence in Research, Kidney. CH, University of Zurich, Zurich, Switzerland; Department of Medicine and Cell physiology and Metabolism, University of Geneva, Geneva, Switzerland; Department of Medicine and Cell physiology and Metabolism, University of Geneva, Geneva, Switzerland; Division of Intensive Care, Department of Acute Medicine, Geneva University Hospitals, Geneva, Switzerland; Department of Medicine and Cell physiology and Metabolism, University of Geneva, Geneva, Switzerland; Service of Clinical Pathology, Department of Pathology and Immunology, University Hospitals and University of Geneva, Geneva, Switzerland; Department of Medicine and Cell physiology and Metabolism, University of Geneva, Geneva, Switzerland; Department of Medicine and Cell physiology and Metabolism, University of Geneva, Geneva, Switzerland; Service of Clinical Pathology, Department of Pathology and Immunology, University Hospitals and University of Geneva, Geneva, Switzerland; Department of Medicine and Cell physiology and Metabolism, University of Geneva, Geneva, Switzerland; Department of Medicine and Cell physiology and Metabolism, University of Geneva, Geneva, Switzerland; Department of Chemistry, University of Zurich, Zurich, Switzerland; Department of Chemistry, University of Zurich, Zurich, Switzerland; Biomaterials Science Center, Department of Biomedical Engineering, University of Basel, Allschwil, Switzerland; Biomaterials Science Center, Department of Biomedical Engineering, University of Basel, Allschwil, Switzerland; Micro- and Nanotomography Core Facility, Department of Biomedical Engineering, University of Basel, Allschwil, Switzerland; III Department of Medicine, University Medical Center Hamburg-Eppendorf, Hamburg, Germany; Nephrological Center, Medical Clinic and Polyclinic IV, University of Munich, Munich, Germany; Department of Medical Physiology, Texas A&M University Health Science Center, Bryan, TX, USA; Service of Clinical Pathology, Department of Pathology and Immunology, University Hospitals and University of Geneva, Geneva, Switzerland; Institute of Physiology, University of Zurich, Zurich, Switzerland; National Centre of Competence in Research, Kidney. CH, University of Zurich, Zurich, Switzerland; Institute of Physiology, University Medicine Greifswald, Greifswald, Germany; Institute of Physiology, University of Zurich, Zurich, Switzerland; National Centre of Competence in Research, Kidney. CH, University of Zurich, Zurich, Switzerland; Department of Medicine and Cell physiology and Metabolism, University of Geneva, Geneva, Switzerland; Service of Nephrology, Department of Medicine, Geneva University Hospitals, Geneva, Switzerland; National Centre of Competence in Research, Kidney. CH, University of Zurich, Zurich, Switzerland

**Keywords:** asparaginyl hydroxylase, CKD, FIH, HIF, HIF1AN, hypoxia

## Abstract

**Background:**

The roles of hypoxia and hypoxia inducible factor (HIF) during chronic kidney disease (CKD) are much debated. Interventional studies with HIF-α activation in rodents have yielded contradictory results. The HIF pathway is regulated by prolyl and asparaginyl hydroxylases. While prolyl hydroxylase inhibition is a well-known method to stabilize HIF-α, little is known about the effect asparaginyl hydroxylase factor inhibiting HIF (FIH).

**Methods:**

We used a model of progressive proteinuric CKD and a model of obstructive nephropathy with unilateral fibrosis. In these models we assessed hypoxia with pimonidazole and vascularization with three-dimensional micro-computed tomography imaging. We analysed a database of 217 CKD biopsies from stage 1 to 5 and we randomly collected 15 CKD biopsies of various severity degrees to assess FIH expression. Finally, we modulated FIH activity *in vitro* and *in vivo* using a pharmacologic approach to assess its relevance in CKD.

**Results:**

In our model of proteinuric CKD, we show that early CKD stages are not characterized by hypoxia or HIF activation. At late CKD stages, some areas of hypoxia are observed, but these are not colocalizing with fibrosis. In mice and in humans, we observed a downregulation of the HIF pathway, together with an increased FIH expression in CKD, according to its severity. Modulating FIH *in vitro* affects cellular metabolism, as described previously. *In vivo*, pharmacologic FIH inhibition increases the glomerular filtration rate of control and CKD animals and is associated with decreased development of fibrosis.

**Conclusions:**

The causative role of hypoxia and HIF activation in CKD progression is questioned. A pharmacological approach of FIH downregulation seems promising in proteinuric kidney disease.

## Graphical Abstract

**Graphical Abstract:**
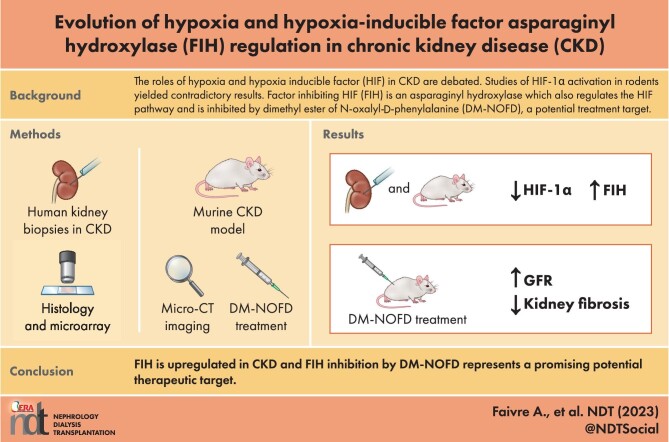

KEY LEARNING POINTS **What is already known about this subject?**

- The pathogenic role of hypoxia and hypoxia-inducible factor (HIF) activation during chronic kidney disease (CKD) is currently debated.

**What this study adds?**

- This study assesses the presence of hypoxia in a model of proteinuric kidney disease and reveals that HIF-1α is not upregulated in different CKD models as previously suggested.
- We show that the asparaginyl HIF hydroxylase, the factor inhibiting HIF (FIH), is upregulated in CKD.
- We further test an inhibitor of FIH, the dimethyl ester of N-oxalyl-D-phenylalanine (DM-NOFD), in conditions of CKD and show a potential protective effect.

**What impact this may have on practice or policy?**

- This study offers new evidence to support the paradigm shift about hypoxia in CKD.
- Moreover, we showed that FIH inhibition in a mouse model of proteinuric CKD could improve renal function and slow down fibrosis development. This could represent a new therapeutic target in the field of CKD treatment.

## INTRODUCTION

Chronic kidney disease (CKD) is endemic worldwide and causes major morbidity, mortality and health system costs [1]. Currently, most CKD cases are secondary to an initial glomerular injury leading to proteinuria [2]. Focal segmental glomerulosclerosis (FSGS), either primary or as a secondary lesion, is a leading cause of proteinuric kidney disease [3]. Proteinuria in turn is a major indicator of CKD progression [4]. The pathogenesis of CKD progression following the initial injury is much debated; in particular, the role of hypoxia and hypoxia inducible factor (HIF) transcription factors in CKD progression has been investigated for several years.

Fine and Norman [5] presented the hypothesis that hypoxia is one of the major events causing a vicious circle of CKD progression. This hypothesis was based on the observed loss of peritubular capillaries in CKD, leading to alterations of perfusion and oxygenation. However, since tubular cells are the major consumers of oxygen within the kidney, their atrophy and the described tubular loss of mitochondria could also decrease oxygen consumption within the kidney and balance the effects of capillary rarefaction.

The HIF pathway may play pro-inflammatory and profibrotic roles in fibrosis [6]. In this regard, genetic HIF-α downregulation seems overall protective in several models of CKD [7]. However, HIF-α activation is also debated in CKD. The expression of classical HIF targets, such as vascular endothelial growth factor, is lower rather than increased during CKD [8], which is not in agreement with an increase in HIF activity. In diabetic nephropathy, some studies even showed decreased HIF-α activation, possibly via increased inflammation and direct inhibitory effects of glucose; in these studies, HIF-α activation prevented CKD progression [9–11]. The recently developed HIF prolyl hydroxylase inhibitors (HIF-PHIs) are not only able to correct renal anaemia in clinical settings [12, 13], but preclinical data indicate a protective role in various murine models of CKD [14–16], even though this is questioned in the clinical setting [14]. Taken together, in animal models, continuous activation of the pathway by genetic upregulation appears detrimental. In contrast, transient pharmacological stimulation may be protective, which may also be related to the absence of cell specificity of the pharmacological stimulation [7].

Besides prolyl hydroxylases, which regulate HIF-α protein levels, the HIF pathway is also regulated by an asparaginyl hydroxylase called factor inhibiting HIF (FIH) [15–17]. Recently, this hydroxylase was shown to be involved in several likely HIF-independent metabolic processes, such as mitochondrial biogenesis, glucose metabolism and inflammation [18, 19]. A global inhibition of all HIF hydroxylases, including FIH, with L-mimosine or cobalt chloride was protective in various CKD models [20, 21]. However, a more specific inhibition of FIH has not yet been tested in such models.

In this study, we use a murine model of progressive CKD following the apoptosis of podocytes. This model displays all the characteristics of CKD from a glomerular origin and mimics FSGS [22]. In this article we assess the local presence of hypoxia, the renal microvascularization and HIF-1α induction during the progression of CKD in experimental models and in kidney biopsy databases from CKD patients, as well as the expression of FIH. Then we modulate FIH activity with a pharmacologic FIH inhibitor, dimethyl ester of N-oxalyl-D-phenylalanine (DM-NOFD; Sigma-Aldrich, St. Louis, MO, USA), experimentally and show a protective role of the compound on CKD progression.

## MATERIALS AND METHODS

### Animals

All animal studies were approved by the Institutional Ethical Committee of Animal Care in Geneva and cantonal authorities (animal authorization numbers GE-70/19, GE-181/19 and GE14920A). Animals had free access to standard diet and water and were housed at 20°C. The proteinuric model of CKD (POD-ATTAC) was obtained as previously described [22]. Briefly, POD-ATTAC male mice on FVB background were injected with dimerizer (Clontech Laboratories, Mountain View, CA, USA) at 8 weeks. After preparation according to manufacturer's datasheet, the dimerizer was injected intraperitoneally, either 0.5 μg/g once or 0.2 μg/g each day during 5 consecutive days. Mice were euthanized after 7 days or 28 days, respectively. Littermate non-transgenic mice were used as controls and injected with the dimerizer as well. DM-NOFD (Sigma-Aldrich) was resuspended according to manufacturer's instructions in dimethyl sulfoxide (DMSO; Thermo Fisher, Waltham, MA, USA), diluted in 0.9% sodium chloride (NaCl) and injected at a dosage of 200 mg/kg intraperitoneally during 28 consecutive days. Controls were injected with an equivalent volume of DMSO diluted in 0.9% NaCl. Unilateral urinary tract obstruction was performed as described previously [23, 24]. We used 8-week-old male mice on a C57BL/6J background. After buprenorphine analgesia and isoflurane anaesthesia, the left ureter of each mouse was ligated; the right kidneys, non-obstructed, were used as controls. Organ collection occurred 7 days after the surgery.

### Renal function assessment

Glomerular filtration rate (GFR) was measured transcutaneously by measuring the excretion rate of fluorescein isothiocyanate sinistrin (Fresenius Kabi, Bad Homburg, Germany) with a minicamera (MediBeacon, St. Louis, MO, USA) as described previously [24]. Filtration was calculated with the appropriate formula [25] in millilitres per minute per kilogram of body weight, then normalized to controls and expressed as a fold change.

### Kidney perfusion

The POD-ATTAC mice received a terminal injection of pentobarbital (Inresa, Bartenheim, France). The superior mesenteric artery, coeliac artery, abdominal artery and inferior vena cava were isolated and ligated, then the vena cava and the aorta were catheterized. A PrismaSol 4-based solution enriched with 10% haematocrit and appropriate electrolytes was used. This solution was oxygenated with a mix of oxygen (95%) and carbon dioxide (5%) at a flow rate of 2 l/min through a membrane oxygenator (Medos Hilite, Medos Medlizintechnik, Stolberg, Germany). The oxygen partial pressure was maintained at ≈30 kPa in the perfusion solution. Maximal oxygen consumption measurement was performed after 20 min of kidney flushing and 40 min of perfusion at 350 μl/min using an ABL90 Flex analyser (Radiometer Medical, Brønshøj, Denmark).

### Cell culture

mpkCCD (RRID:CVCL_R771), mCCD_cl1_ [26] and immortalized human proximal tubular HK-2 cells (ATCC CRL-2190; American Type Culture Collection, Manassas, VA, USA) were used and cultured as described previously [27, 28]. Cells were transfected with plasmids expressing human wild-type FIH-V5 or small interfering RNA (siRNA) targeting human FIH as described previously [29, 30], during 24 hours. Empty vectors or control siRNAs were transfected in parallel. The sequences for siRNAs are provided in Supplementary Table 1. The oxygen consumption rate of the cells was measured using a Seahorse XF96 Analyser and Seahorse XFp Cell Mito Stress Test Kit (103010-100; Agilent Technologies, Santa Clara, CA, USA) according to manufacturer's instructions.

### Erythropoietin measurement

The erythropoietin levels in the plasma were measured using the Quantikine enzyme-linked immunosorbent assay (ELISA) kit (MEP00B; R&D Systems, https://www.rndsystems.com/products/mouse-erythropoietin-epo-quantikine-elisa-kit_mep00b) according to manufacturer’s instructions. Briefly, the blood was collected by intracardiac sampling and centrifuged at 2000 *g* for 10 min. The supernatant was collected and diluted to half before the ELISA.

### Histology and immunohistochemistry

Kidneys were fixed in 4% paraformaldehyde (Alfa Aesar, Ward Hill, MA, USA), paraffin embedded and a 5-μm section was cut with a microtome. To perform fibrosis quantification, we proceeded as described previously [24]. Briefly, a median section of the kidney was selected and stained with Sirius Red solution (Abcam, Cambridge, UK), according to the manufacturer's protocol. The slides were scanned with an Axioscan image scanner (Zeiss, Oberkochen, Germany) at 20× magnification. The cortical area was manually defined and red-stained areas were automatically quantified by Tissue Phenomics software (Definiens, Munich, Germany). Immunostainings were performed using a citrate buffer (10 mM, pH 6) microwave-based antigen target retrieval technique and the EnvisionFlex kit from Dako (Santa Clara, CA, USA). The antibodies used are listed in Supplementary Table 1. Detection of local tissue hypoxia was performed using a Hypoxyprobe-1 Kit (HP1-1000, Hypoxyprobe, Burlington, MA, USA). Mice received an intraperitoneal injection of Hypoxyprobe-1 at a dosage of 60 mg/kg in saline 60 min before euthanasia. The immunostaining of pimonidazole adducts in formalin-fixed, paraffin-embedded tissue was performed according to the method described below.

### Western blotting

Kidney tissue samples were homogenized in 100 μl or 1 ml in cold lysis buffer [20 mmol/l Tris hydrochloride (Tris-HCl), 2 mmol/l ethylenediaminetetraacetic acid, 30 mmol/l sodium fluoride, 30 mmol/l sodium pyrophosphate, 0.5 mol/l sodium orthovanadate, 20% sodium dodecyl sulphate (SDS), 10% Triton-X-100 and Roche cOmplete mini protease inhibitor mixture) on ice. Proteins were prepared in solution containing Tris-HCL 50 mM, glycerol 10%, SDS 1%, bromophenol blue 0.01% and 2-mercaptoethanol 10 mM, then heated for 5 min at 95°C. A protein concentration assay was performed using both a Pierce BCA Protein Assay (Thermo Scientific, Waltham, MA, USA) and Coomassie Blue gel staining (Thermo Scientific GelCode blue stain reagent). A total of 25 μg of proteins were loaded in 10% bis-acrylamide homemade gels and migration was performed at 100 V for 1 hour. Transfer was made on nitrocellulose membrane (GE Healthcare, Chicago, IL, USA) for 1 hour at 100 V. After washes in TBS-Tween 0.1% (Tris-HCL 50 mM, sodium chloride 150 mM and Tween 20 0.1%) and blocking at room temperature in 5% milk–TBS–Tween for 1 hour, the membrane was incubated overnight at 4°C with primary antibody diluted in 5% milk–TBS–Tween. After washing, the membrane was then incubated for 1 hour with goat anti-rabbit horseradish peroxidase (HRP) secondary antibody (1:5000) or goat anti-mouse HRP secondary antibody (1:10 000). All antibody references are provided in Supplementary Table 1. Protein expression was detected with enhanced chemiluminescence detection reagent WesternBright Quantum (Advansta, San Jose, CA, USA), whose chemiluminescence was revealed with a PXi gel imaging system (Syngene, Frederick, MD, USA). Band density was quantified using ImageJ software (National Institutes of Health, Bethesda, MD, USA). Protein expression was normalized to Ponceau staining. Results are expressed as the fold change in protein expression compared with the control samples.

### Human kidney biopsy immunohistochemistry and immunofluorescence

Human kidney biopsies were collected within the Service of Pathology from Geneva University Hospitals. Tissues were fixed in formaldehyde, embedded in paraffin and cut in 3-μm-thick sections. A total of 14 samples were randomly selected and included in this study, with various renal disease and degrees of renal fibrosis and renal function. Renal fibrosis was graded after standard analyses by an experimented renal pathologist. Sections were incubated with a polyclonal rabbit anti-FIH antibody (1:100), as described previously [31]. Slides were digitized and graded in terms of FIH expression by two nephrologists in a blinded fashion. Each patient provided informed consent before inclusion in the study. The institutional ethics committee approved the clinical protocol (CEREH 03-081). The research was performed according to the Helsinki Declaration principles.

For immunofluorescence, 12 biopsies from 12 CKD patients and 3 non-injured human kidneys (autopsy cases) were sliced at 4 μm and incubated all together (one batch experiment) overnight at 4°C with HIF-AN polyclonal antibody (ab187524, 1/50 dilution) followed by 1 hour of incubation at room temperature with secondary immunoglobulin G CY3 conjugated antibody (AB2338000, 1/200 dilution). The nucleus was stained with 4′,6-diamidino-2-phenylindole for 10 min at room temperature (1/1000) and mounted with Vectashield (Vector Laboratories, Newark, NJ, USA). After staining, all slices were scanned with the same setting on a widefield Axioscan Z1 scanner (Zeiss) at 20× magnification. The renal cortex was annotated in QuPath version 0.4.2 excluding glomeruli and a detection of nuclei was performed using StarDist. Then a random tree (RTrees) was trained to classify interstitial, tubular negative and tubular positive cells in the 12 biopsies and 3 control kidneys. The number of positive tubular cells was expressed as a ratio of total tubular cells.

### Microarray data analyses of human kidney biopsies

Published Affymetrix microarray expression data from the European Renal cDNA–Kröner-Fresenius Biopsy Bank (ERCB-KFB; CKD: GSE 99340, Living Donors: GSE32591, GSE35489, GSE37463) were analysed for messenger RNA (mRNA) expression levels of HIF hydroxylases. Sample collection, RNA isolation and preparation and microarray analysis were performed as described previously [32]. Biopsies from different kidney diseases were used [cadaveric donor (CD), tumour nephrectomy (TN), diabetic nephropathy (DN), thin basement disease (TMD), minimal change disease (MCD), hypertensive nephropathy (HTN), immunoglobulin A nephropathy (IgAN), FSGS, membranous nephropathy (MGN), lupus nephritis (LN) and anti-neutrophil cytoplasmic antibody vasculitis (AAV) and grouped into different CKD stages (CKD 1–5) according to their estimated GFR (eGFR) calculated by the Chronic Kidney Disease Epidemiology Collaboration (CKD-EPI) equation [33]. Pretransplantation kidney biopsies from healthy living donors served as the control group. To identify differentially expressed genes, the significance analysis of microarrays (SAM) method was applied using TiGR MultiExperiment Viewer version 4.9 [34]. A *q*-value <5% was considered to be statistically significant.

### Real-time quantitative polymerase chain reaction (qPCR)

Total RNA from cells and kidney tissue samples was extracted with Trizol reagent (Invitrogen, Waltham, MA, USA) or with the RNA extraction kit (Machery-Nagel, Hoerdt, France) according to the manufacturer's instructions. RNA concentration and purity were measured using the NANODROP 2000C Spectrophotometer (Thermo Scientific) and 1 μg of total RNA was reverse transcribed using qScript cDNA supermix (Quanta Biosciences, Beverly, MA, USA). Complementary DNA was used to perform qPCR in triplicate using PowerUp SYBR Green Master Mix (Applied Biosystems, Waltham, MA, USA) and StepOne Plus Real-Time PCR System (Applied Biosystems) or a QuantStudio 5 Real-Time PCR System (Thermo Fisher Scientific). The 2^−ΔΔCT^ method was used to analyse the relative changes in gene expression levels. Primers used in qPCR are listed in Supplementary Table 1.

### Micro-computed tomography (CT) imaging and analysis

Mice were perfused with the in-house developed X-ray contrast agent XlinCA. Sample preparation and analysis were performed as described previously [35]. XlinCA was synthesized as described previously [36–38]. After anaesthesia with ketamine/xylazine, kidneys were perfusion-fixed via the abdominal aorta and then injected with XlinCA contrast agent. The abdominal aorta was cannulated with a blunted 21-gauge butterfly needle, then the abdominal aorta and mesenteric artery were ligated. A window was cut into the vena cava as an outlet, then the kidneys were flushed with 10 ml of phosphate-buffered saline (PBS) to remove blood, then fixed with 100 ml of 4% formaldehyde/1% glutaraldehyde in PBS at 37°C with 150 mmHg pressure. To avoid premature crosslinking, 20 ml of PBS and then 50 ml of glycine solution (5 mg/ml in PBS) were used to remove and quench residual aldehydes. Kidneys were then flushed again with 40 ml of PBS. Three millilitres of XlinCA contrast agent dissolved in water (75 mg iodine/ml) were injected with a pressure-actuated syringe, then 4% glutaraldehyde in PBS was dripped into the abdominal cavity for crosslinking. After gelation, kidneys were removed and immersed in 15 ml of 4% glutaraldehyde/PBS. Before scanning, the kidneys were embedded in 1% agar in PBS in standard 1.5 ml centrifugation tubes.

Kidneys were scanned at 4.444 μm voxel size using a Phoenix Nanotom m X-ray micro-CT (General Electric, Boston, MA, USA) with a tungsten target, 90 kV acceleration voltage and 200-μA beam current. For each of the 1440 projections, nine frames with a 0.5-sec exposure time were recorded and averaged, resulting in a scan time of approximately 6 hours for the three height steps required per kidney. Three-dimensional (3D) volumes were reconstructed using the manufacturer's proprietary Phoenix datoslx software (General Electric). Image processing was performed in a blinded fashion on a workstation equipped with 256 GB RAM and 32-core AMD 3970X Threadripper processor and a Samsung 860 EVO S-ATA SSD. Datasets were 3D Gauss-filtered (σ = 1 pixel), manually thresholded to extract the blood vessels and exported as a stack of two-dimensional TIFF images using Fiji/ImageJ software [39, 40]. They were then further processed using the commercial XamFlow software (LucidConcepts, Zurich, Switzerland). The largest connected component was extracted from the thresholded binary masks to remove particles of contrast agent on the outside of the kidneys. The signed distance transform of the resulting blood vessel segment was then calculated. A mask of the kidney outer shape was created using morphological closing with 50 pixels (corresponding to 222.2 μm) and applied to the signed distance transform. All these morphological operations were performed using the CLIP library, which is capable of leveraging the full parallel processing power of the workstation. For quantification, the cumulative distribution function of the calculated distance values were calculated and evaluated at 20 μm and 100 μm using an unpaired, two-tailed Student's *t*-test implemented in R (R Foundation for Statistical Computing, Vienna, Austria).

### Single-cell RNA sequencing (scRNAseq) data analysis

scRNAseq data were downloaded from https://doi.org/10.5281/zenodo.4059315. Data were further pre-processed using the standard Seurat workflow. Briefly, we performed, for each sample, normalization and variance stabilization with the sctransform v2 function of Seurat. We thus applied the RunPCA, RunUMAP, FindNeighbors and FindClusters functions with default parameters. To compare gene expressions among conditions, we used the PrepSCTFindMarkers and FindMarkers functions. Finally, we used PROGENy to infer pathways activity from gene expression.

### Statistics

Group statistics were analysed by *t*-test and two-way analysis of variance/Bonferroni's multiple comparison test for two or three groups, respectively. Results are expressed as the fold change compared with controls unless specified otherwise.

## RESULTS

### Early stages of proteinuric CKD are not characterized by hypoxia or HIF activation

**Figure 1:**
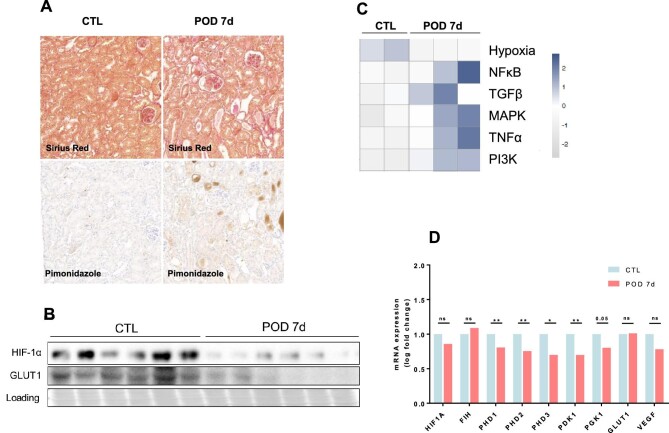
Early stages of proteinuric CKD are not characterized by hypoxia and HIF activation. **(A)** Representative serial sections of control (CTL) and POD-ATTAC mice 7 days after a single dose of dimerizer injection (POD 7d). In the upper panel, Sirius Red staining and in the lower panel, pimonidazole immunohistochemistry are shown. **(B)** Representative immunoblot of kidney cortex in CTL (*n* = 6) and POD 7d (*n* = 6) mice for HIF-1α and GLUT1. **(C)** RNA sequencing analysis in CTL (*n* = 2) and POD 7d mice (*n* = 3) and corresponding heatmap of PROGENy pathways (hypoxia, NF-κB, TGFβ, MAPK, TNFα, PI3K). **(D)** mRNA expression assessed by qPCR in CTL (*n* = 6) and POD 7d (*n* = 6) of HIF-1α, FIH, PHD1, PHD2, PHD3, PDK1, PGK1, GLUT1 and vascular endothelial growth factor. Results are presented as the fold change compared with controls, with error bars showing mean ± standard deviation. *P < .05, **P < .01.

Single-dose dimerizer injection led to proteinuria, modest GFR decline and inflammation, as previously described [28], without marked fibrosis (Fig. 1A). Hypoxia was not detected by pimonidazole staining in the early CKD progression stages (Fig. 1A). Protein expression of HIF-1α was decreased, as well as one of its targets, GLUT1 (Fig. 1B). In RNA sequencing analysis, the global state of activation of the hypoxia pathway was assessed using the hypoxia PROGENy pathway [41], which was also globally downregulated, together with an increase in the classically activated profibrotic and pro-inflammatory pathways (Fig. 1C), suggesting that HIF activation is not an early event in CKD. qPCR analysis confirms a decreased expression of HIF-α target genes despite a downregulation of prolyl-4-hydroxylase (PHD) expression (Fig. 1D).

### In late stages of proteinuric CKD, vascularization is rarefied but does not induce general tissue hypoxia

**Figure 2:**
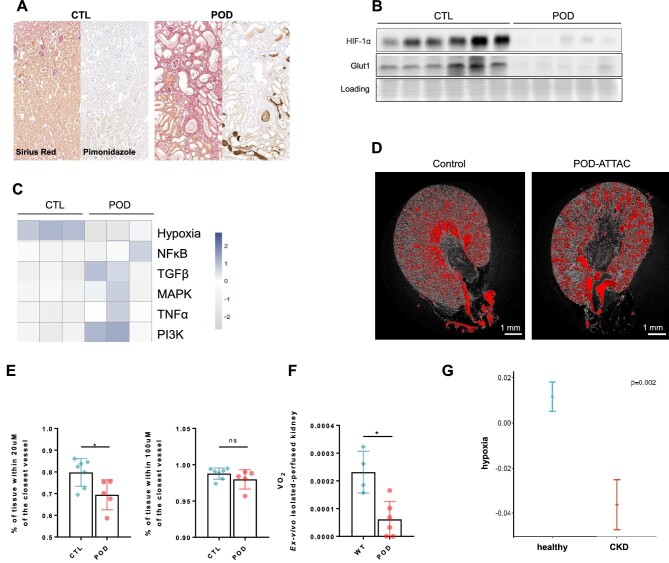
In late stages of proteinuric CKD, vascularization is rarefied but does not induce major hypoxia. **(A)** Representative serial sections of control (CTL) and POD-ATTAC mice 28 days after repeated injections of dimerizer (POD). In the left panel, Sirius Red staining and in the right panel, pimonidazole immunohistochemistry. **(B)** Representative immunoblot of kidney cortex in CTL (*n* = 6) and POD (*n* = 5) mice for HIF-1α and GLUT1 and corresponding loading control (Ponceau S staining). **(C)** RNA sequencing analysis in CTL (*n* = 3) and POD mice (*n* = 3) and corresponding heatmap of PROGENy pathways (hypoxia, NF-κB, TGFβ, MAPK, TNFα, PI3K). **(D)** Representative visualization of renal microvascularization assessed by micro-CT imaging following contrast agent perfusion. **(E)** Percentage of kidney tissue within 20 μm or 100 μm of the closest blood vessel, quantified by micro-CT following contrast agent perfusion, in CTL (*n* = 7) and POD (*n* = 5) kidneys. *P < .05. **(F)** In the *ex vivo* perfused kidney setup, renal oxygen consumption (VO_2_) in CTL (*n* = 4) and POD (*n* = 6) kidneys. *P < .05. **(G)** Hypoxia PROGENy pathway in human FACS sorted PDGFRb-positive analysed by scRNAseq indicating a global deactivation of the pathway in CKD (*P* = .002). Results are presented with error bars showing mean ± standard deviation.

After the injection of repeated small doses of dimerizer, mice developed classic features of CKD within 28 days, with decreased measured GFR and albuminuria [24]. We further observed extensive fibrosis and tubular atrophy (Fig. 2A). Pimonidazole staining indicated the presence of hypoxia in some minor zones in already advanced CKD, but these areas were overall rare and did not colocalize with fibrotic areas (Fig. 2A and Supplementary Fig. 1). These areas were located in zones of preserved tubular morphology, in which the intact proximal tubular cells likely consume more oxygen than the atrophic cells in fibrotic tissue. Intratubular protein casts present a non-specific staining, which should not be considered. We also observed a downregulation of HIF-1α protein and its target GLUT1, which was even more severe than at early stages (Fig. 2B and Supplementary Fig. 2A). Like in early stages, the hypoxia PROGENy pathway was downregulated and pro-inflammatory or profibrotic pathways were increased (Fig. 2C). We confirmed by qPCR the downregulation of the pathway (Supplementary Fig. 2B). In this model, we previously confirmed that tubular cell number was not decreased [31], precluding decreased tubular mass as an explanation for these observations. We assessed renal vascularization by micro-CT after contrast agent perfusion, as described previously [35]. We observed a decrease in vascular bundles, mainly in the inner stripe of the outer medulla, but less in the cortex, of POD-ATTAC mice (Fig. 2D). A certain amount of capillary rarefaction was present (Fig. 2D, E and Supplementary Fig. 2C); however, 98% of the analysed kidney tissue was close enough (100 μm) to vessel to be within the oxygen diffusion limit (Fig. 2E).

In a model of *ex vivo* isolated perfused kidney [31], oxygen consumption decreased with CKD (Fig. 2F). Therefore, given the decreased consumption and a capillary rarefaction that did not reach the oxygen diffusion limit, most areas of the kidney are likely non-hypoxic in CKD, as we observed with pimonidazole staining. To ensure that the global downregulation of the HIF pathway was not model specific, we repeated the protein expression analysis in the classical unilateral urinary tract obstruction model (UUO) and observed similar results (Supplementary Fig. 3). Finally, in a recent database of single-nuclei RNA sequencing in biopsies from CKD patients, we observed a global decreased of the hypoxia PROGENy pathway in proximal tubular cells (Fig. 2G and Supplementary Fig. 4), confirming that the HIF pathway is not activated, but rather downregulated, during CKD.

### Asparaginyl hydroxylase FIH is upregulated during CKD

**Figure 3:**
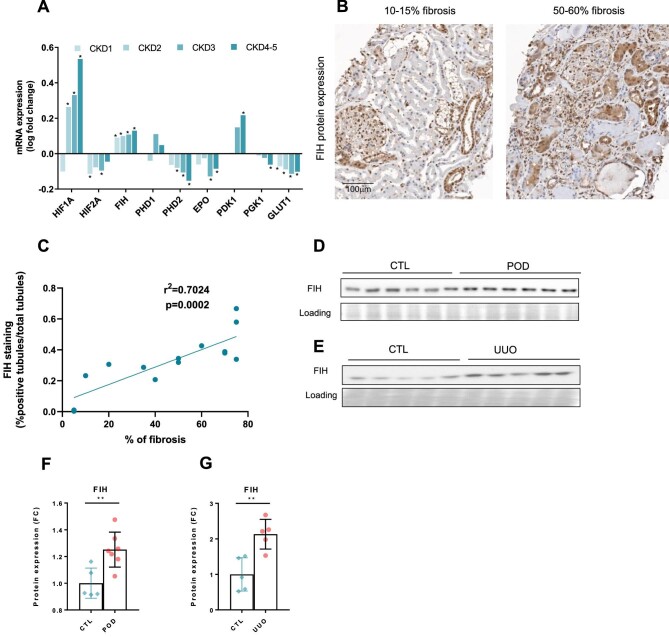
The asparaginyl hydroxylase FIH is upregulated during CKD. **(A)** Analysis of *HIF1A, HIF2A, FIH, PHD1, PHD2, PHD3, EPO, PDK1, PGK1* and *GLUT1* in the Affymetrix microarray expression dataset obtained in the ERCB-KFB, sorted by CKD stage (CKD1, *n* = 56; CKD2, *n* = 46; CKD3, *n* = 37; CKD4, *n* = 26; CKD5, *n* = 10). Biopsies from kidney donors (*n* = 42) are used as controls. *q < 0.05. **(B)** Representative immunostaining of FIH in human kidney biopsies according to fibrosis level assessed by an experienced renal pathologist (left panel: 10–15% of fibrosis; right panel: 50–60% of fibrosis). **(C)** Automated quantification of FIH staining with QuPath and relative correlation with eGFR by creatinine using the CKD-EPI equation in kidney biopsies from 15 CKD patients (fibrosis <20%, *n* = 5; 40–50%, *n* = 5 and >70%, *n* = 4), fitted through linear regression (*r*^2^ = 0.7024). **(D)** Representative immunoblot of kidney cortex in CTL (*n* = 6) and POD (*n* = 5) mice for FIH and corresponding loading control (Ponceau S staining). **(E)** Representative immunoblot of kidney cortex in contralateral kidneys (CTL, *n* = 6) and in kidneys after 5 days of unilateral urethral obstruction (UUO, *n* = 5) for FIH and corresponding loading control (Ponceau S staining). **(F)** Protein expression quantification of kidney cortex in CTL (*n* = 6) and POD (*n* = 5) mice for FIH and corresponding loading control (Ponceau S staining). **(G)** Protein expression quantification of kidney cortex in contralateral kidneys (CTL, *n* = 6) and in kidneys after 5 days of unilateral urethral obstruction (UUO, *n* = 5) for FIH and corresponding loading control (Ponceau S staining). **P < .01. Results are presented with error bars showing mean ± standard deviation.

In order to understand the downregulation of HIF target genes despite some areas of hypoxia, we further assessed the expression of HIF hydroxylases. We observed that FIH was upregulated according to CKD severity in the Kröner-Fresenius databank of 217 CKD biopsies, while prolyl hydroxylases were either not regulated or downregulated (Fig. 3A). In the same cohort, we observed a global decrease in HIF-α target genes *EPO, PGK1* and *GLUT1*. HIF-1α mRNA was upregulated, probably as a compensatory mechanism [42]. We confirmed the upregulation of FIH protein expression in 15 consecutive random CKD biopsies from the Geneva University Hospitals Clinical Pathology Department (Fig. 3B). Automated quantification of FIH expression was correlated with kidney function loss (Fig. 3C and Supplementary Fig. 5A–C) and the staining was strong in proximal tubular cells during CKD (Supplementary Fig. 5D). In our murine models, FIH was also upregulated at the protein level (Fig. 3D–G).

### FIH is an important metabolic regulator *in vitro*

**Figure 4:**
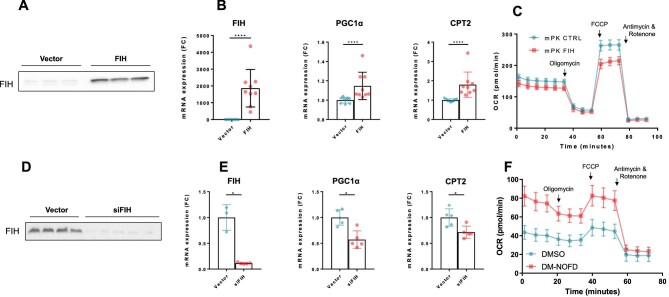
FIH is an important metabolic regulator *in vitro*. **(A)** Representative immunoblot of FIH in mCCD-cl1 cells transfected with control plasmid (vector, *n* = 3) or plasmid encoding for human FIH (FIH, *n* = 3). **(B)** mRNA expression assessed by real-time qPCR of FIH, PGC1α and CPT2 in mCCD-cl1 cells transfected with control plasmid (vector, *n* = 9) or plasmid encoding for human FIH (FIH, *n* = 9). ****P < .0001. **(C)** Oxidative stress test showing the oxygen consumption rate of mpkCCD cells transfected with control plasmid (mPK CTRL) or plasmid encoding for human FIH (mPK FIH) and subjected to oligomycin, carbonyl cyanide-p-trifluoromethoxyphenylhydrazone (FCCP) and antimycin/rotenone. **(D)** Representative immunoblot of FIH in HK-2 cells transfected with control siRNA (vector, *n* = 4) or siRNA targeting FIH (siFIH, *n* = 4). **(E)** mRNA expression assessed by real-time qPCR of FIH, PGC1α and CPT2 in HK-2 cells transfected with control siRNA (vector, *n* = 5) or siRNA targeting FIH (siFIH, *n* = 5). *P < .05. **(F)** Oxidative stress test showing the oxygen consumption rate of primary cultured tubular cells injected 72 hours prior to the experiment with DM-NOFD (DM-NOFD) or vehicle (DMSO) and subjected to oligomycin, carbonyl cyanide-p-trifluoromethoxyphenylhydrazone (FCCP) and antimycin/rotenone. Results are presented with error bars showing mean ± standard deviation.

As FIH is upregulated according to CKD severity, we hypothesized that FIH could play a direct pathogenic role in CKD progression. FIH is a known metabolic regulator [19], which we confirmed in the mouse-derived kidney tubular cells (mpkCCD) cell line. We found that FIH overexpression (Fig. 4A) influenced the regulation of *PGC1α* and *CPT2*, genes involved in mitochondrial biogenesis, mitochondrial function and lipid metabolism (Fig. 4B). It also affects the mitochondrial reserve capacity of mpkCCD cells (Fig. 4C), as previously described in other cell types [43]. We found opposite results with FIH silencing using siRNA in the same kidney cell line (Fig. 4D, E), confirming that FIH also affects cellular metabolism in renal cells. Finally, we isolated cortical cells after 72 hours in DM-NOFD-treated mice and found an increased oxygen consumption rate globally (Fig. 4F), confirming an important metabolic role of FIH in the kidney.

### Pharmacologic inhibition of FIH in proteinuric CKD

**Figure 5:**
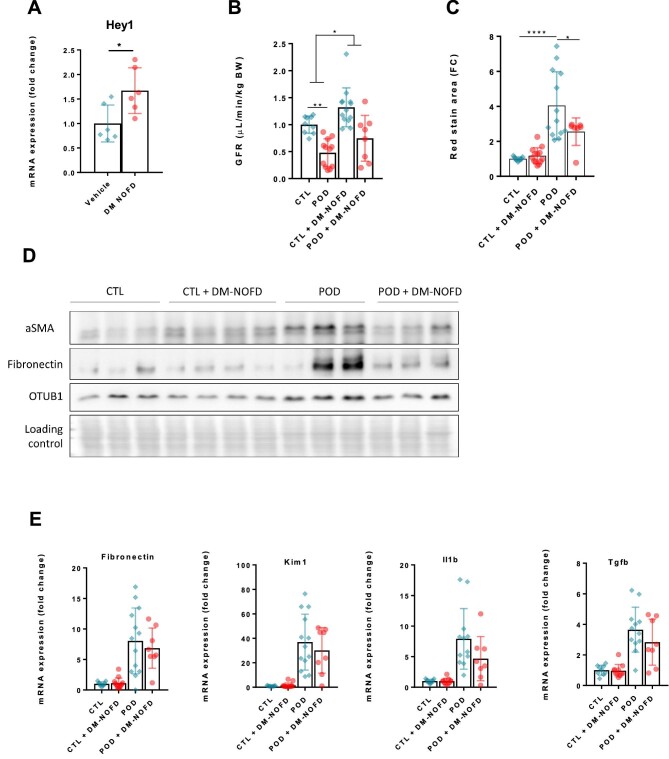
Pharmacologic inhibition of FIH in proteinuric CKD. **(A)** mRNA expression assessed by real-time qPCR of Hey1 in kidney cortex of mice treated with intraperitoneal injections of vehicle (DMSO 0.002%, *n* = 6) or with 200 mg/kg of DM-NOFD. *P < .05. **(B)** GFR assessed by sinistrin clearance in control mice treated with vehicle (CTL, *n* = 9), POD-ATTAC mice 28 days after repeated dimerizer injections and treated with vehicle (POD, *n* = 12), control mice treated with 200 mg/kg of DM-NOFD (CTL + DM-NOFD, *n* = 13) and POD-ATTAC mice 28 days after repeated dimerizer injections and treated with 200 mg/kg of DM-NOFD (POD + DM-NOFD, *n* = 8). *P < .05. **(C)** Quantification of Sirius Red staining in CTL (*n* = 9), POD (*n* = 12), CTL + DM-NOFD (*n* = 13) and POD + DM-NOFD (*n* = 8). *P < .05, ****P < .0001. **(D)** Representative immunoblot of kidney cortex in CTL (*n* = 9), POD (*n* = 12), CTL + DM-NOFD (*n* = 13) and POD + DM-NOFD (*n* = 8) for α-SMA, fibronectin and OTUB1 with corresponding loading control (Ponceau S staining). **(E)** mRNA expression assessed by real-time qPCR in CTL (*n* = 9), POD (*n* = 12), CTL + DM-NOFD (*n* = 13) and POD + DM-NOFD (*n* = 8) of fibronectin, KIM-1, IL-1b and TGFb genes.

DM-NOFD is a small molecule inhibitor targeting preferentially FIH [44, 45]. Its inhibitory effect on FIH is well-described *in vitro*, but has, to our knowledge, not been tested in animals before as a whole-body application. We found that a regimen of 200 mg/kg every 24 hours for 28 days efficiently inhibited a downstream target of the Notch pathway, Hey1 (Fig. 5A), without affecting erythropoietin levels (Supplementary Fig. 6A). In our model of proteinuric CKD, DM-NOFD globally increases the GFR of mice in the POD-ATTAC and control group (Fig. 5B). This increase in GFR is not accompanied by an increased albuminuria (Supplementary Fig. 6B). We also observe an alleviation of fibrosis development, as assessed by Sirius Red staining and quantification (Fig. 5C) and Western blotting of fibronectin and α-smooth muscle actin (Fig. 5D). Although an important variability is present, preventing a statistical significance, we observe a global tendency to alleviate pro-inflammatory and profibrotic markers by qPCR as well (Fig. 5E and Supplementary Fig. 6C).

## DISCUSSION

Hypoxia has been described as a major mechanism causing the progression of CKD [5, 46]. This hypothesis has been proposed because of the observation of decreased vascularization in progressive renal disease. However, this hypothesis does not consider the important role of tubular cells as energy consumers in the kidney. Indeed, the metabolic state of cells largely influences local oxygen consumption and therefore HIF pathway activation. Decreased vascularization, although present, may not lead to oxygen deficiency in all kidney areas, given the fact that energy demand decreases in parallel in areas of tubular atrophy. Using a model of progressive proteinuric CKD, we show that hypoxia is not present in the early stages of CKD; in late stages, hypoxia is preferentially localized in remnant nephrons rather than in fibrotic zones. High-definition 3D imaging of kidney microvasculature revealed a loss of capillaries mainly in the inner stripe of the outer medulla; however, 98% of the analysed kidney tissue was close enough (100 μm) to a vessel to be within the oxygen diffusion limit of healthy tissue. We note that stating this limit presumes unchanged diffusion characteristics between healthy and diseased tissue. While it is conceivable that the limit may be altered in disease, we are not aware of reliable *in vivo* data on oxygen diffusion coefficients for fibrotic renal tissue.

Besides hypoxia, the role of the HIF pathway in CKD has been debated and interventional studies in CKD models have yielded mixed results. Genetic modulations of HIF expression indicate a detrimental role, whereas pharmacological modulations show overall a protective effect [7]. In diabetic nephropathy, insufficient HIF-1α activation was proposed as a factor of progression [47], and the efficiency of HIF-PHIs in renal anaemia treatment also points towards suboptimal activation [13]. In our models, we show that HIF-1α protein levels and its pathway are generally decreased, in both the early and late stages of CKD, despite a paradoxical downregulation of classical PHD expression. This was observed in two animal models as well as in two datasets of biopsies from CKD patients. The cause of this downregulation is unclear and does not seem dependent on classical PHDs, which were themselves downregulated. FIH is a key regulator of HIF transcription, known to be expressed at baseline in the distal nephron, where it may avoid continuous activation of the HIF pathway [48]. In murine models and in human CKD, FIH was largely overexpressed in early and advanced CKD [16], especially in the proximal tubules and interstitial cells. Although other regulators of HIF may still participate in the observed HIF inhibition, the overexpression of FIH is expected to play an important role in HIF pathway inhibition, as well as metabolism and inflammation regulation.

FIH has been described as a key regulator of metabolism, affecting especially lipid and mitochondrial metabolism, via modulations of the adenosine monophosphate–activated protein kinase and peroxisome proliferator-activated receptor gamma co-activator 1α (PGC1α) pathways, among others [19, 29, 49]. In cells, the modulation of FIH expression affected genes involved in fatty acid oxidation and mitochondrial biogenesis, such as PGC1α, and functionally influenced mitochondrial respiration. Mitochondrial dysfunction and downregulation of fatty acid oxidation are major factors leading to CKD progression, as reviewed elsewhere [50]. A direct pathogenic role of FIH in CKD progression is therefore possible.

As HIF pathway modulation had a differential effect on CKD progression depending on the approach used, with a more favourable effect expected with pharmacologic modulations, we tested *in vivo* a specific FIH inhibitor, DM-NOFD. To our knowledge, this specific compound has not been tested *in vivo* before. In mice, 200 mg/kg of DM-NOFD every 24 hours intraperitoneally efficiently inhibited FIH protein expression and increased the GFR at baseline, an effect similar to the protective knock-in of PGC1α, that could be mediated by the metabolic properties of FIH, which we show *in vitro* and *in vivo* by Seahorse analysis [51]. The exact cause of the enhancement of GFR at baseline is unclear and no albuminuria was observed. In our model of proteinuric CKD, DM-NOFD also tended to improve the loss of renal function in terms of GFR and fibrosis development. Altogether, our results indicate that DM-NOFD efficiently inhibits FIH *in vivo*, is well tolerated and could be protective in a model of proteinuric CKD. Studies with more animals and morphological histology data are needed to confirm our hypothesis.

In summary, we show that hypoxia is not an early phenomenon in CKD and is mainly located in remnant nephrons. The HIF pathway is generally downregulated during CKD and increased FIH expression may participate in this regulation. Finally, inhibiting FIH preferentially may play a protective role in CKD progression.

## Supplementary Material

gfad075_Supplemental_File

## Data Availability

The data underlying this article will be shared upon reasonable request to the corresponding author.
