# Supplementary material for: Evolution of hypoxia and hypoxia-inducible factor asparaginyl hydroxylase regulation in chronic kidney disease
Source: Nephrol Dial Transplant. 2023 Apr 24;38(10):2276–88. doi: 10.1093/ndt/gfad075 (PMC10539236; doi:10.1093/ndt/gfad075)
Supplement: gfad075_Supplemental_File [file gfad075_supplemental_file.pptx]

## Slide 1
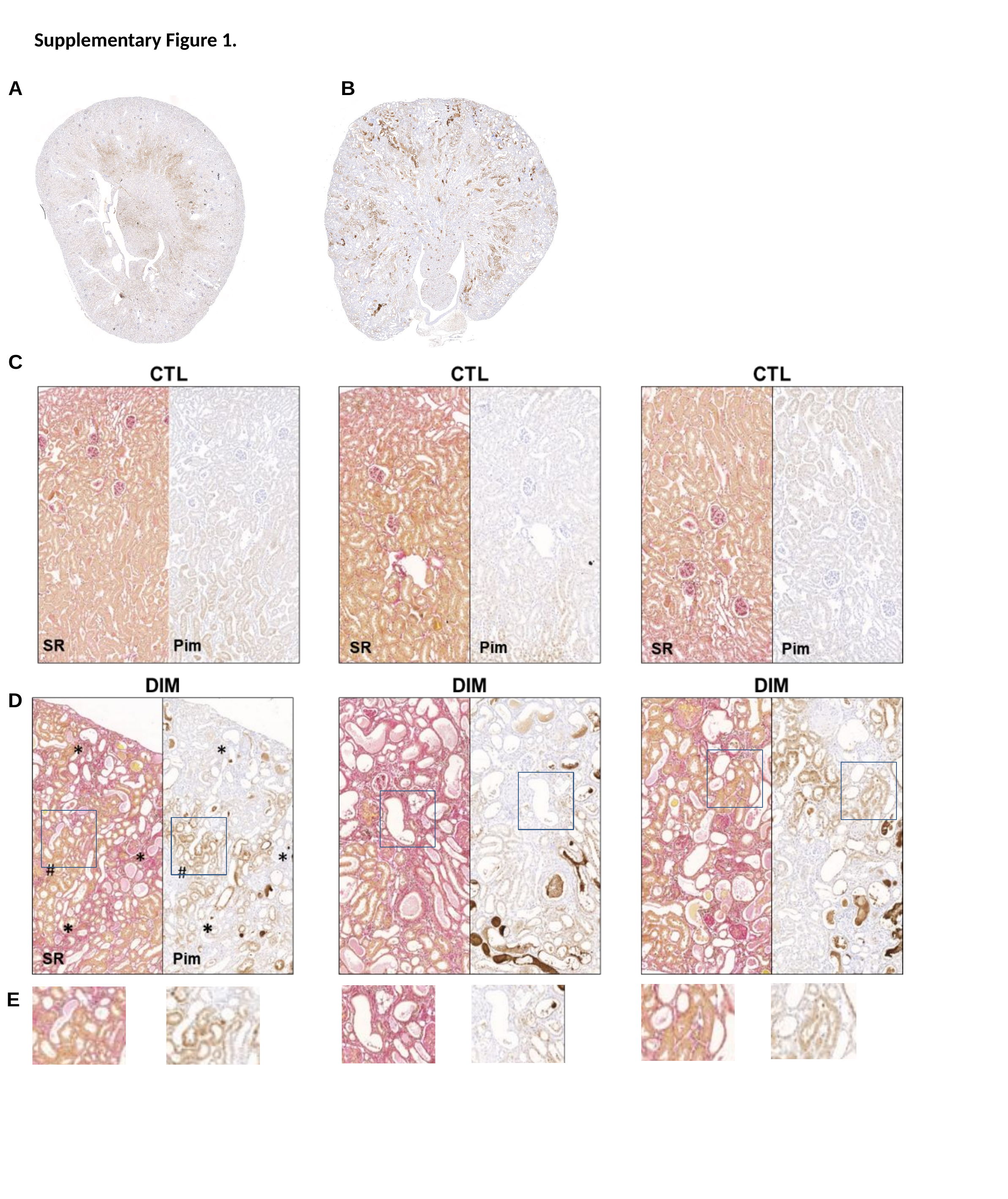

Supplementary Figure 1.
A
B
C
D
E

## Slide 2
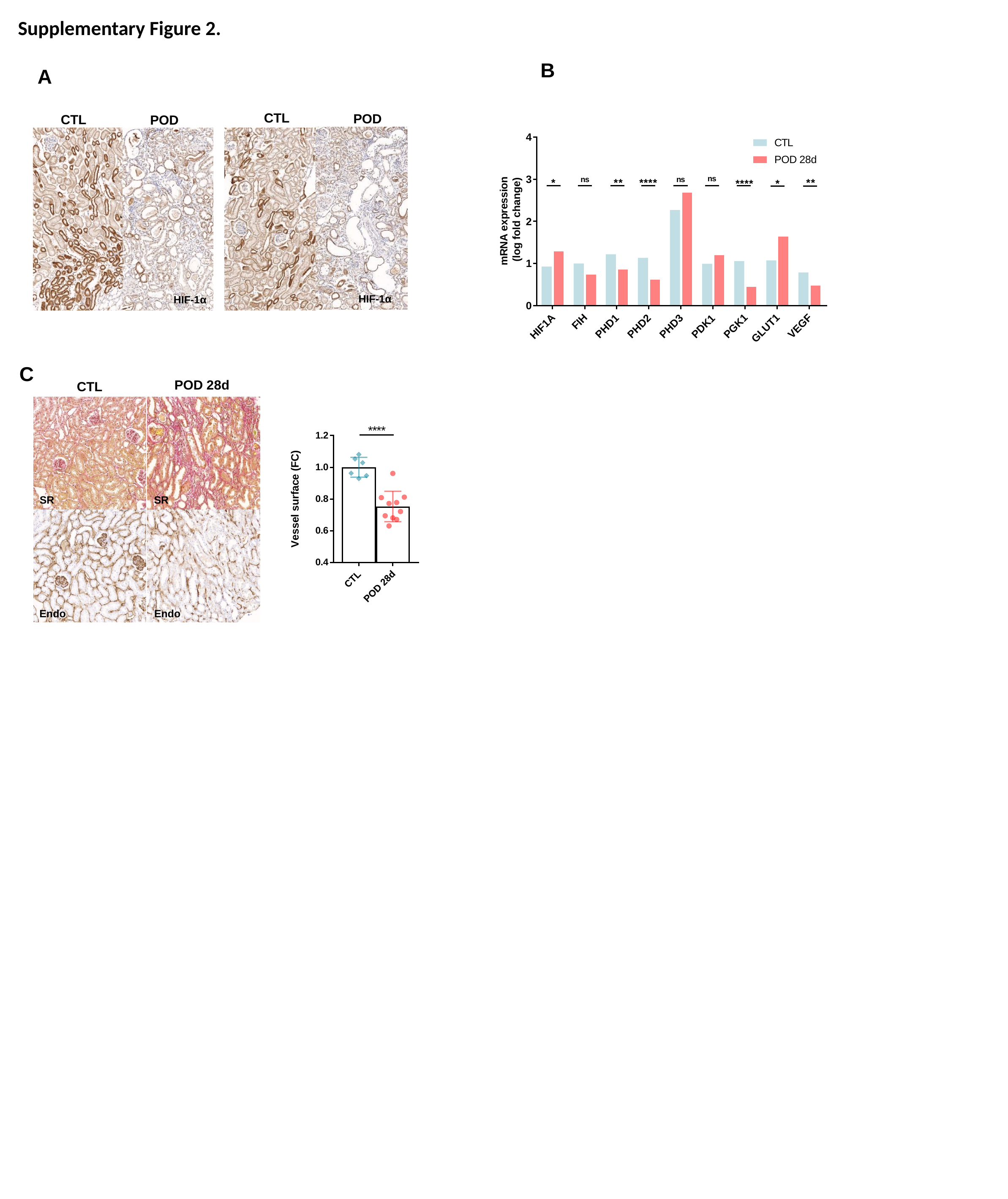

Supplementary Figure 2.
B
A
CTL
POD
CTL
POD
HIF
HIF-1α
HIF-1α
C
POD 28d
CTL
SR
Endo
SR
Endo

## Slide 3
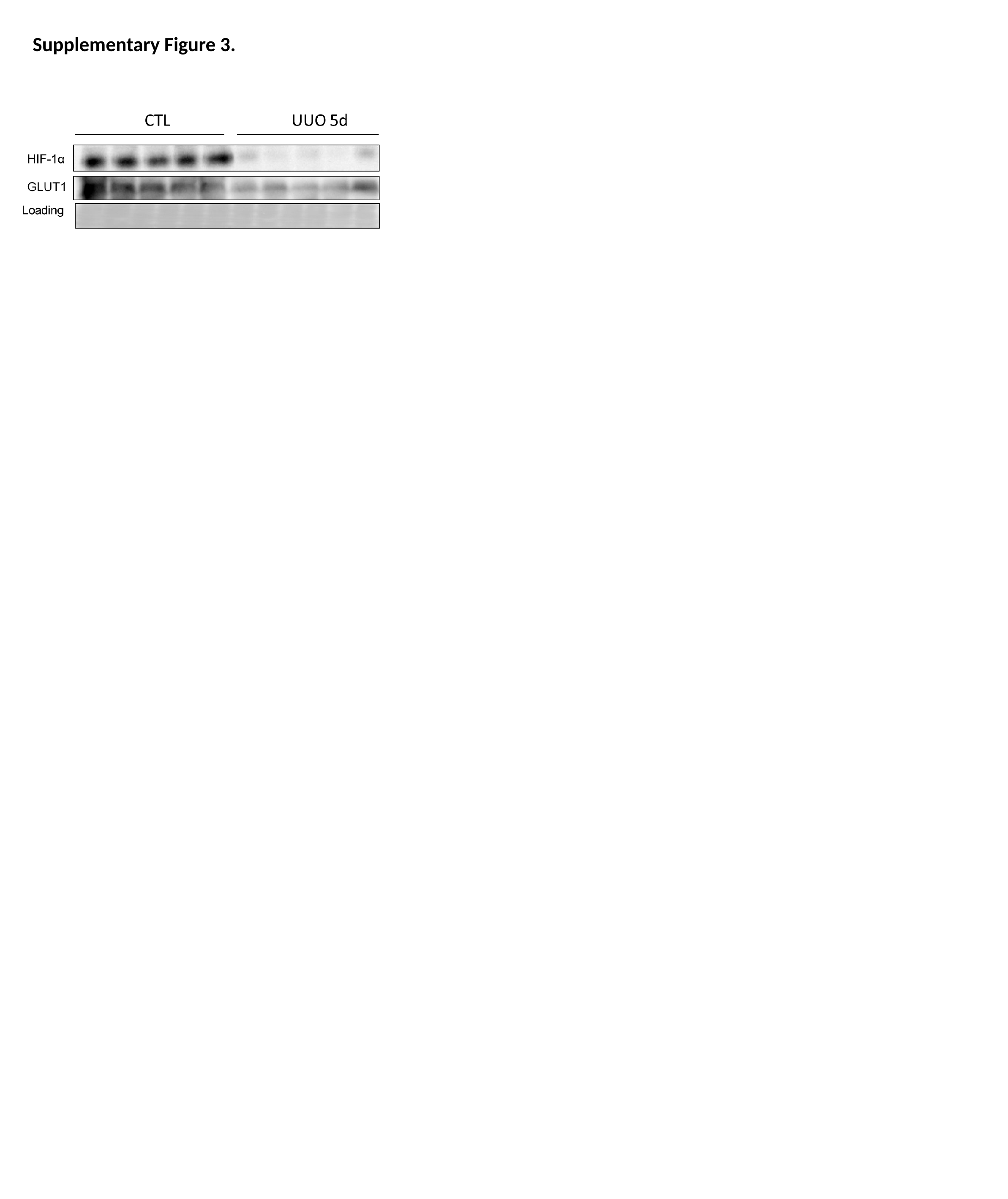

Supplementary Figure 3.

## Slide 4
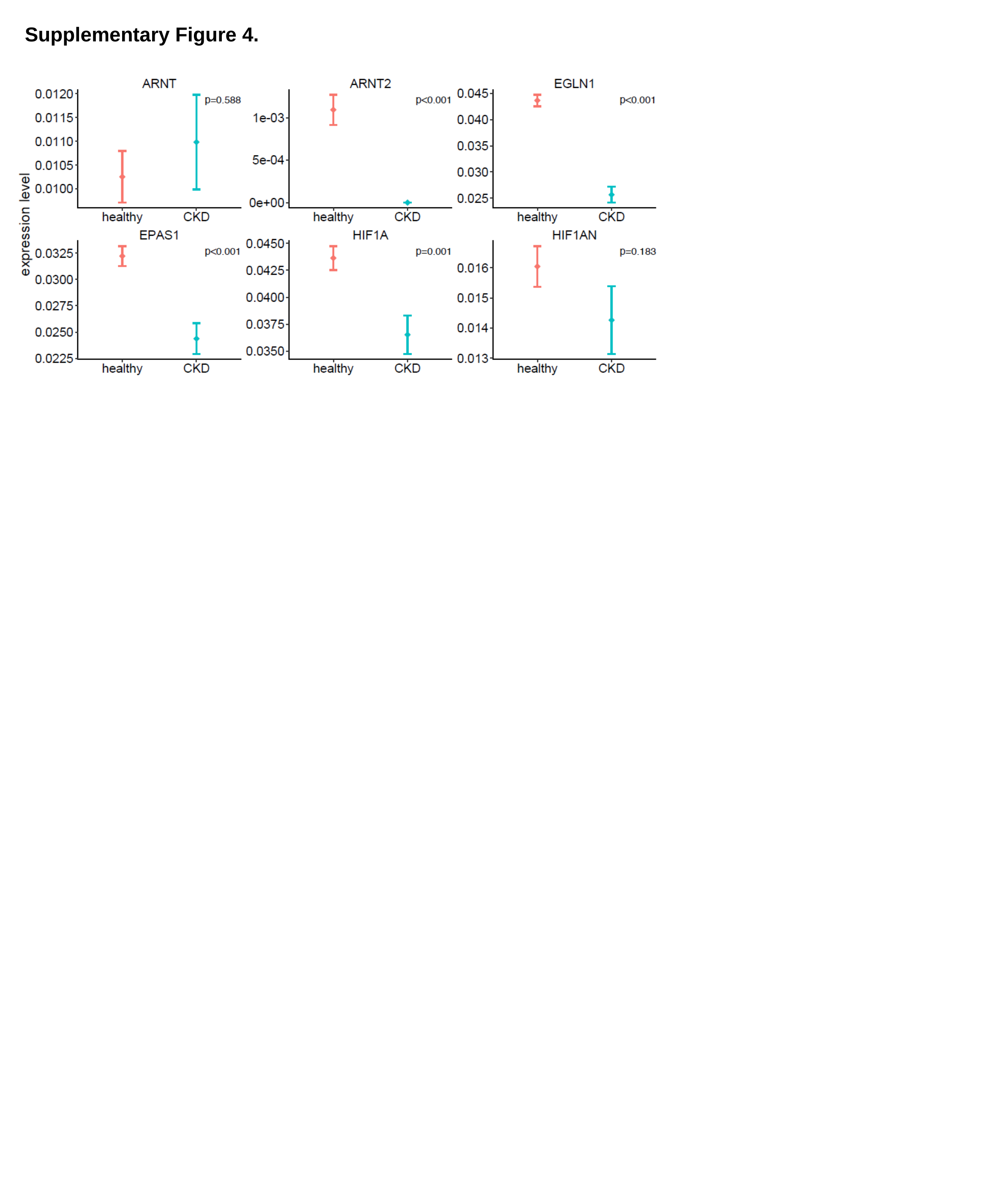

Supplementary Figure 4.

## Slide 5
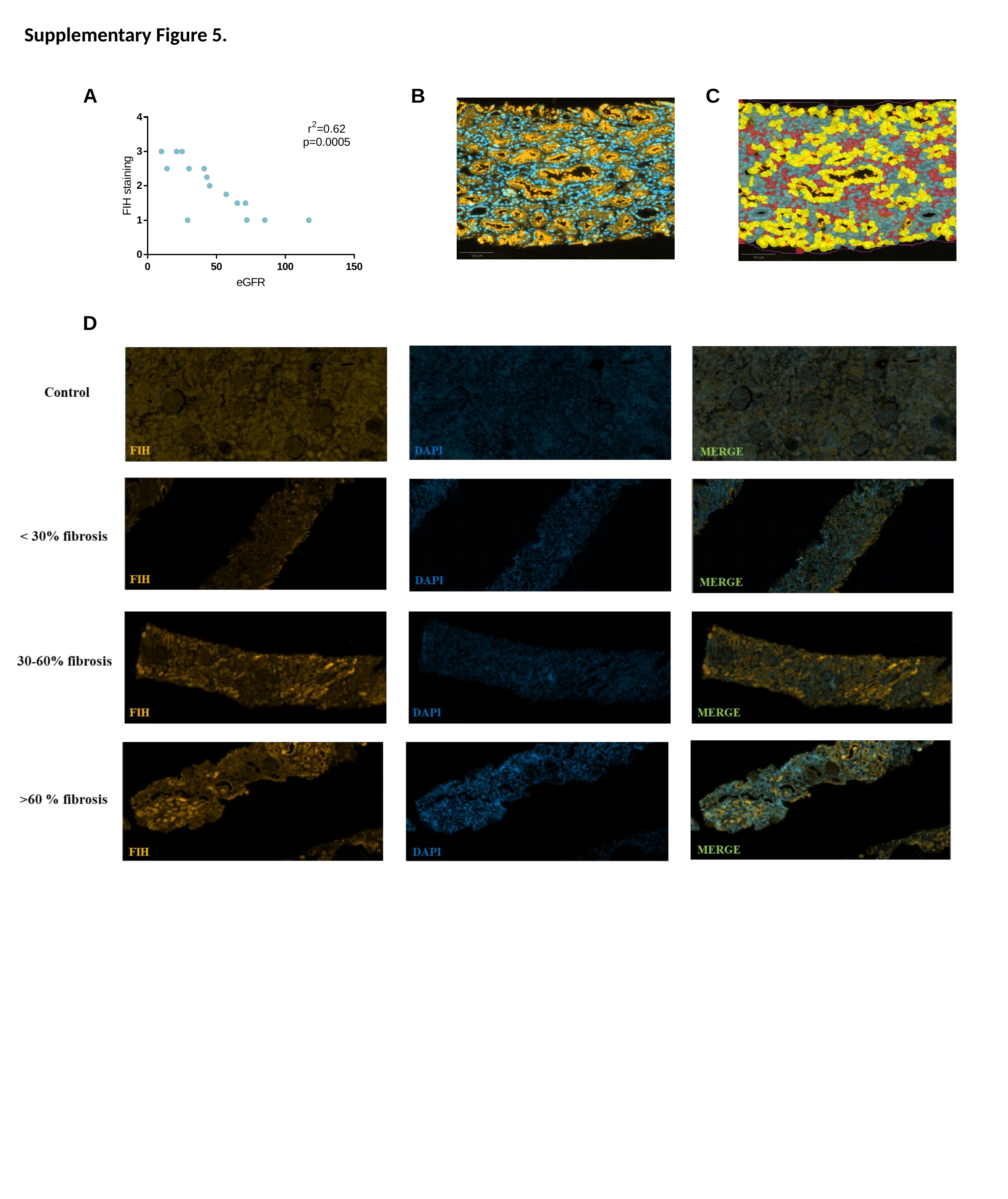

Supplementary Figure 5.
B
C
A
D

## Slide 6
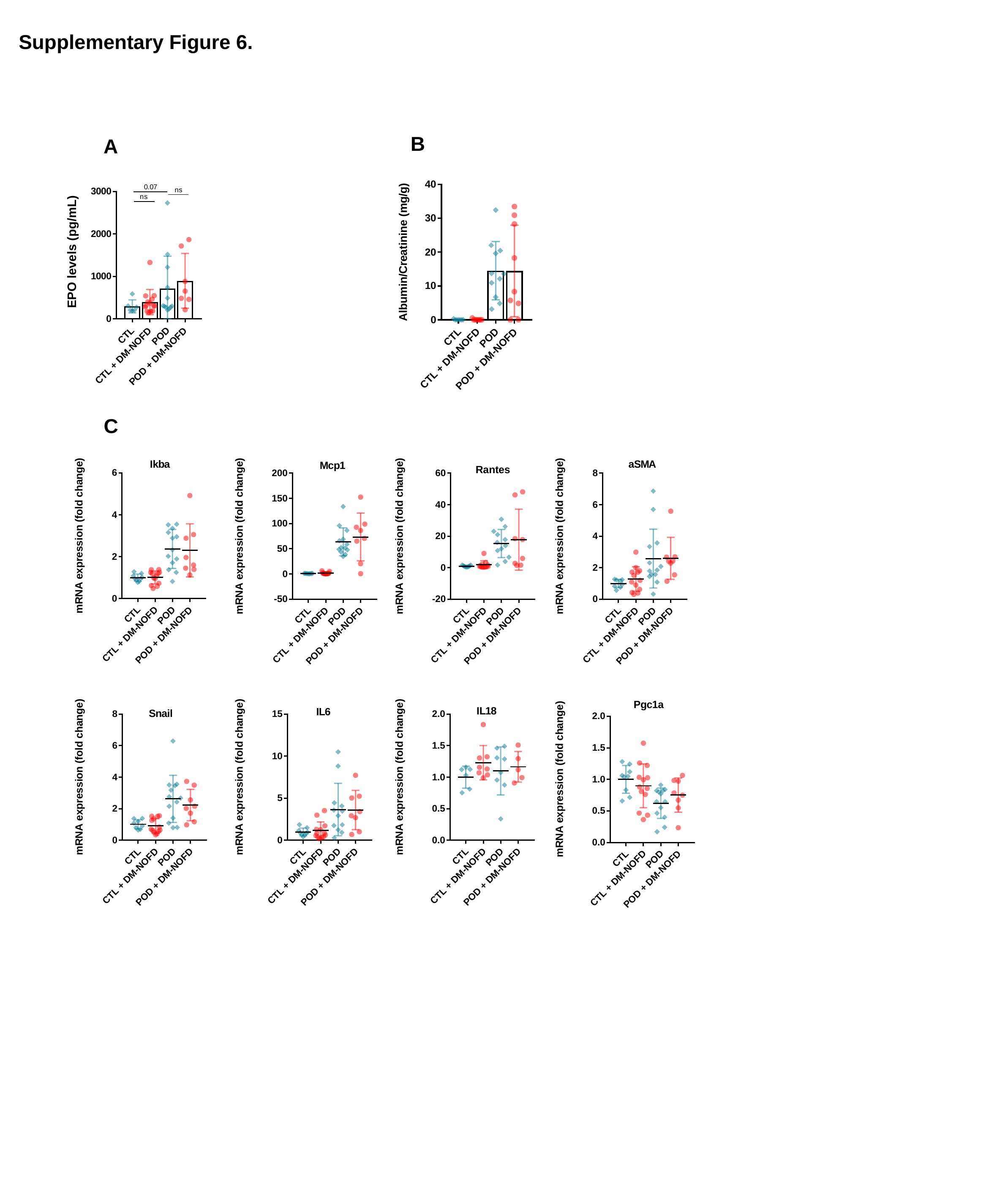

Supplementary Figure 6.
B
A
EPO levels (pg/mL)
C
